# Fluoride Retention following the Professional Topical Application of 2% Neutral Sodium Fluoride Foam

**DOI:** 10.1155/2011/209349

**Published:** 2011-07-07

**Authors:** Wenqun Song, Shinji Toda, Eri Komiyama, Karin Komiyama, Yuki Arakawa, Dawei He, Hirohisa Arakawa

**Affiliations:** Division of Oral Health, Department of Health Science, Kanagawa Dental College, Inaokacho 82, Yokosuka 238-8580, Japan

## Abstract

The objective of the present research was to determine the appropriate amount of fluoride to use professional topical application and the residual amounts of fluoride in the oral cavity using a 2% neutral sodium fluoride (NaF) foam with a dedicated tray. Using dentition study models, a method for determining the appropriate amount of NaF foam was investigated. In eight adult subjects, the appropriate amount of NaF foam, the concentration of fluoride in the saliva following professional topical application, and the amount of residual fluoride in the oral cavity following professional topical application was examined. The results indicated that the appropriate amount of NaF foam was approximately 0.8 g, the amount of residual fluoride in the oral cavity was approximately 25% of the amount of foam used.

## 1. Introduction

Methods of topical fluoride application include professional topical application and home use of fluoride dentifrice and fluoride mouth rinse. Professional topical application is a means of preventing dental caries in both deciduous and permanent teeth and is performed by dental specialists (dentists and dental hygienists) in dental clinics, health care facilities, and other appropriate settings specializing in dental care. Advantages of professional topical fluoride application include the ability to treat patients starting from a very young age, immediately after the deciduous anterior teeth erupted, in addition to the fact that application is necessary only two to four times a year. In addition, the ingestion of fluoride from the professional topical fluoride application is not considered to be a risk factor in dental fluorosis [1]. The fluoride agents in professional topical application are available in liquid, gel, and foam types. Because the gel and foam are generally applied using a tray, these have an advantage in that the fluoride can be applied to all of the teeth simultaneously [2]. In the United States and a number of other countries, the tray method using acidulated phosphate fluoride (APF) foam is widely used, and the foam application procedure, including the amount of foam used, is standardized [3]. In Japan, the Ministry of Health and Welfare has issued “Guidelines Governing Professional Topical Application of Fluoride,” which describes a method for applying the neutral and acidic NaF liquids that were initially introduced in Japan in the 1960s [4]. A method for applying APF gel, which was subsequently introduced in the 1980s, has also been established based on fundamental research on factors, such as the amount of gel to be used and the residual amount in the oral cavity [5–9]. Strongly acidic APF has an advantage in that a large amount of uptake into the tooth substance can be expected, but there are also reports expressing concerns about damage occurring to several types of restorative material [10–15]. These reports underscore the necessity for topical application of neutral NaF, and as a result, neutral NaF foam is now available on the market in Japan. However, no information is currently available on aspects such as methods for applying this neutral NaF foam or the amount of foam that remains in the oral cavity after application. 

## 2. Materials and Methods

### 2.1. Experiment  1: Investigation of Appropriate Amount of NaF Foam Using Dentition Study Models

First, various amounts of foam (Butler Fluodent Foam N, Sunstar Inc., Osaka, Japan) in the deciduous and permanent dentition study model trays (Butler Tray, Sunstar Inc., Osaka, Japan) were prepared to apply to the deciduous and permanent dentition study models (Study model PE-ANA004 and Study model PE-ANA002, respectively, Nissin Dental Products Inc., Kyoto, Japan) [5]. The trays were the accompanying items of the 2% neutral NaF foam. They were made of styrene foam, unlined type. Initially, the weight of tray without the foam was measured by an electronic balance (LIBROR AEG-45SM, SHIMAZU Co., Kyoto, Japan), then the foam was placed, and the total weight was measured. The amount of fluoride foam was calculated by subtraction of the weight of tray from the total weight. 

To sufficiently cover all the dried teeth surfaces of the study models and to minimize leakage outside of the dentition, the appropriate amount of foam (AAF) was filled to be adjusted to a level approximately 2 mm below the tray. Using the deciduous and permanent dentition study models, the foam was applied for a 4-minute period. After the foam application, the tray was removed from the study model immediately, and the net weight of foam remained in the tray (TRF: tray-remained foam) and the net weight foam adhering to the study model were measured, respectively. Then the foam adhering the area of mucosa of study model was wiped with a wiping paper (Kimwipe S-200, NIPPON PAPER CRECIA Co., LTD, Tokyo, Japan ), and the net weight of foam adhering to the surface of study model (TRSF: tooth-surface retained foam) was measured. The weight difference between above study models was defined as overflowed foam (OF). Each of these was measured 10 times.

### 2.2. Experiment  2: Changes over Time in Fluoride Concentration in Saliva following NaF Foam Application

Based on the procedure of experiment 1, an application experiment was carried out in human subjects. Eight healthy participants (three males and five females), ranging in age from 21.3 to 24.8 years, were recruited from the students at Kanagawa Dental College. The mean (±SD) age of the subjects was 22.6 ± 1.3 years. Each subject signed the informed consent form, which had been approved by the Ethics Committee of Kanagawa Dental College (No. 44). 

The inclusion criteria in this study were: (1) with at least 28 natural teeth; (2) unstimulated saliva flow rate > 0.3 mL/min; (3) no orthodontic appliance in their oral cavity. The exclusion criteria were: (1) clinically detectable caries; (2) periodontitis; (3) history of allergies and metabolic diseases such as diabetes; (4) other medical condition that could interfere with the study.

Subjects were instructed to brush their teeth using a nonfluoride toothpaste starting 3 days before the day of the experiment until the end of the experiment, and all other fluoride applications were suspended. The experiment began at 2:00 p.m. Before the NaF foam was applied to subjects, their teeth were dried with the compressed air. The trays were filled with NaF foam to a level approximately 2 mm below the edge of the trays and were placed over the teeth, the subject was instructed to close the jaws with the trays in contact for 4 minutes. The subject was instructed not to swallow but to allow the saliva to dribble into a 500-mL plastic beaker held directly under the mouth. At the end of the topical application, the trays were removed from the mouth and placed in the same beaker, and the subject expectorated the mixture of saliva and foam into the same beaker immediately after removal of the tray and expectorated once again 30 seconds later. Whole saliva was collected into a separate 50-mL plastic vessel for 5 minutes a total of eight times (immediately prior to application, 5 minutes afterwards, 15 minutes afterwards, 30 minutes afterwards, 60 minutes afterwards, 120 minutes afterwards, before the subjects went to bed, and immediately after they awoke the following day). 

The saliva samples obtained up to 30 minutes afterwards were diluted by double-deionized water at the ratio of 1 : 10, and others were not diluted. Total ionic strength adjustment buffer (TISAB) (TISAB II, Orion Research Inc., Beverly, Mass, USA) were added to all saliva samples as the ratio of 1 : 1, and the fluoride concentration of saliva was analyzed as ppm using the ion-specific electrode (Orion 9609BNWP combination electrode, Thermo Electron Corp., Beverly, Mass, USA).

### 2.3. Experiment  3: Amount of Residual Fluoride in the Oral Cavity following Application of NaF Foam

As in Experiment  2, NaF foam was applied for 4 minutes using the same procedure. The subject was instructed not to swallow but to allow the saliva to dribble into a 500-mL plastic beaker held directly under the mouth. At the end of the topical application, the trays were removed from the mouth and placed in the plastic beaker, and the subject expectorated the mixture of saliva and foam into the same beaker and expectorated once again 30 seconds later. Total amount of 500 mL double-deionized water was added to the beaker. The solution was stirred by the magnetic stirrer until no traces of the foam were visible. Then the solution was analyzed for fluoride concentration using the ion-specific electrode (Orion 9609BNWP combination electrode, Thermo Electron Corp., Beverly, Mass, USA). 

The total amount of fluoride recovered to the oral cavity was calculated as the product of the fluoride concentration and volume. The weight of residual fluoride was calculated by subtracting the weight recovered from the weight applied.

In addition, the ratio of residual fluoride in the oral cavity was determined from the percentage of fluoride remaining in the oral cavity in relation to the amount of fluoride used.

### 2.4. Statistical Analysis

 The data are expressed as mean ± SD. The test of population mean for the fluoride concentrations in saliva following the foam application and the test of coefficient of correlations between with AAF, TRF, TRSF, and OF in the study models were analyzed (JMP, ver. 8, SAS institute Japan, Ltd., Tokyo, Japan). An alpha of 0.5 was selected a priori as the indicator for statistical significance.

## 3. Results

### 3.1. Experiment  1: Investigation of the Appropriate Amount of NaF Foam Using Dentition Study Models

Using deciduous and permanent dentition study models, we found that in order to sufficiently cover the dentition when applying the NaF foam and to minimize leakage outside of the dentition, the tray needed to be filled to a level approximately 2 mm below the tray edge. Using this method, we applied NaF foam 10 times to deciduous and permanent dentition study models. The results showed significant variation in mean appropriate amount of foam (AAF); that of the deciduous dentition study model was 0.57 ± 0.17 (0.36–0.86) g (Table 1), while that of the permanent dentition study model was 0.96 ± 0.24 (0.68–1.39) g (Table 2). The mean of the amount of foam retained on the tooth surfaces (TSRF: tooth-surface-retained foam) of the deciduous and permanent dentition study models were 0.03 ± 0.01 (0.01–0.05) g and 0.12 ± 0.06 (0.03–0.20) g, respectively, (Tables 1 and 2).

Analysis of the coefficient of correlations between the appropriate amount of foam (AAF) and the amount of foam adhering to and recovered from the tray (TRF: tray-retained foam), AAF and the amount of foam adhering to the mucosa other than the tooth surface on the models (OF: overflowed foam), and AAF and the tooth surface retained foam (TSRF) showed a strong positive correlation between AAF and TRF and between AAF and OF in both the deciduous and permanent dentition study models (Table 3).

### 3.2. Experiment  2: Changes over Time in Fluoride Concentration in Saliva following NaF Foam Application

**Figure 1:**
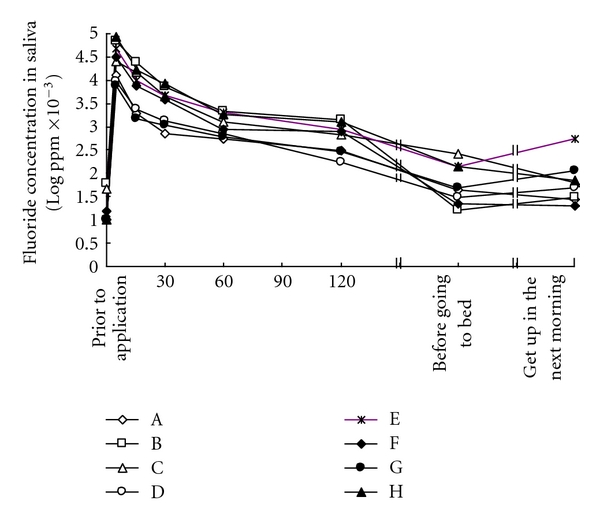
Changes over time in fluoride concentration in saliva following 2% neutral NaF foam application. A–H indicates each subject in this study.

The mean amount of NaF foam applied in each adult subjects was 0.81 ± 0.20 (0.54–1.12) g. The mean fluoride concentration in saliva following application was 35.95 ± 28.84 (7.48–85.80) ppm, which was the maximum value 5 minutes after application, and by 30 minutes after application had dropped sharply. It continued to drop gradually thereafter, but the concentration of fluoride in saliva measured at the time subjects awoke the following morning was relatively high, at 0.12 ppm (Figure 1). There were significantly differences in salivary fluoride concentrations up to 120 min following the application compared with 0.05 ppm as criterion value (test of population mean, *P* < .05).

### 3.3. Experiment  3: Amount of Residual Fluoride in the Oral Cavity following Application of NaF Foam

The mean amount of NaF foam used was 0.80 ± 0.22 (0.56–1.16) g. The mean amount of fluoride used was 7.18 ± 1.94 (5.00–10.47) mg, and the mean amount of fluoride used per kilogram body weight was 0.14 ± 0.04 (0.09–0.19) mg F/kg. The amount of fluoride recovered was 5.44 ± 1.75 (3.65–8.70) mg, and the amount and ratio of residual fluoride in the oral cavity after foam application were 1.74 ± 0.47 (1.32–2.44) mg and 24.9 ± 5.80 (16.9–34.40) %, respectively. The amount of residual fluoride in the oral cavity per kilogram body weight was 0.033 ± 0.012 (0.017–0.053) mg F/kg (Table 4). A strong correlation was observed between the amount of fluoride used per kilogram body weight and the amount of residual fluoride in the oral cavity per kilogram body weight (*r* = 0.71,  *P* < .05).

## 4. Discussion

The fluoride agents in professional topical application are available in liquid, gel, and foam types. Currently, the fluoride agents in professional topical application are available in liquid or gel types in Japan. Accompanying documentation specifies that the amount of professional topical application to be used is 2 mL or less using the paint-on technique [16]. This applies when a liquid or a gel is used but does not apply to foams. 

In an experiment conducted by Whitford et al. [17] in 46 children between the ages of 8 and 12 years, the appropriate amount of APF foam or APF gel used to cover the dentition without leaking into the oral cavity was one-third the depth of the tray, which was 0.89 ± 0.02 g of foam and 3.86 ± 0.06 g of gel. With these results, the amount of foam needed to sufficiently cover the dental surface was 23.1% that of gel. Moreover, in research by Arakawa et al. [5] using APF gel, the amount of gel used with a deciduous dentition study model was 2.13 ± 0.77 g and that for a permanent dentition study model was 3.94 ± 1.38 g, while the amounts of residual gel on the dental surface after wiping away gel that had leaked from the dentition study models were 0.06 ± 0.03 g and 0.18 ± 0.08 g, respectively. In our experiment, we initially prepared enough foam to reach the tray edge, meaning an amount of foam equivalent to the capacity of the tray. When the prepared foam was applied to the study model, a large amount of foam overflowed from the tray and the model. As a result, it was determined that the appropriate amount of foam should be filled to be a level approximately 2 mm below the edge of the tray. The mean amount of foam used with the deciduous dentition study model was 0.57 (0.36–0.86) g (Table 1) and that with the permanent dentition study model was 0.96 (0.68–1.40) g (Table 2). These are 26.7% and 24.9%, respectively, of the amounts of gel reported by Arakawa et al. [5] and are largely consistent with the results reported by Whitford et al. [17]. For the amounts of foam remaining on tooth surfaces, the results were 0.03 (0.01–0.05) g for the deciduous dentition study model and 0.12 (0.03–0.20) g for the permanent dentition study model (Table 4), which was approximately half the amount of gel reported by Arakawa et al. 

In the present experiments, the variations in the appropriate amount of foam were comparatively wide, even though the tray was filled with foam to a level approximately 2 mm below the tray edge, using the same study model and using foam from the same container. The reason of this phenomenon is not clear. 

In Experiments  2 and 3, the tray was filled with foam to a level approximately 2 mm below the tray edge. The mean amount of foam used for the eight adult subjects was approximately the same, 0.80 g. This was largely consistent with the amount of foam used by Whitford et al. [17]. This result suggests that with the neutral NaF foam used in the present experiments, the amount used can be set at a lower amount than that for gel. In research conducted by Sudo et al. [18], in which APF gel was applied using the toothbrush method in subjects 1.5 years of age, the mean amount of gel applied was 0.66 g, and the mean amount of residual fluoride in the oral cavity per kilogram body weight was 0.19 mg/kg. The mean residual ratio was 25.8%. In our experiments, the mean amount of residual fluoride in the oral cavity following application of NaF foam was 1.74 mg, and the amount per kilogram body weight was 0.033 mg/kg, with a mean residual ratio of 24.9% (Table 4). Comparing the results of this study, the residual ratios in our experiments were largely the same, but because our experiments targeted adult subjects, the residual amount of fluoride per kilogram body weight was extremely small. The amount of fluoride that causes acute toxicity is 1.35 to 1.8 mg/kg or higher [19]. The results of our experiment indicate that the maximum value would be 0.16 mg/kg, even in a child weighing 15 kg; therefore, this amount assures adequate safety.

Where the application of highly acidic dental coatings such as APF is cited as possibly causing corrosion of dental restorative materials such as glass-containing resin composites, glass-ionomer cement, and porcelain, 0.9% neutral NaF foam is reported to have little effect on surface hardness of the materials [20, 21]. Moreover, in patients who have restorative materials, use of a neutral NaF coating is recommended in order to avoid corrosion, discoloration, and other problems with the restorative materials [22].

 Earlier studies have reported that when fluoride products such as dentifrice, rinse, and gels are used, a high fluoride concentration in the saliva was initially obtained, subsequently it dramatically reduced with time [23, 24]. The results of this research showed that the concentration of fluoride in saliva immediately following foam application was extremely high and still be elevated at 1.2 ppm F in the next morning (Figure 1). The retention of fluoride in saliva may be very important in the prevention or reversal of caries. It has been proven that low levels of fluoride (0.03–0.5 mg/L) in saliva are sufficient to effectively inhibit demineralisation and/or enhance remineralisation of enamel [25]. It seems that this 2% neutral NaF foam is effective in prevention of caries immediately after the application because the salivary fluoride concentration up to 120 min is higher than 0.05 ppm.

Based on the results of the research described here, using a 2% neutral NaF foam enables application using the tray method with smaller amounts of fluoride. Another advantage that follows is that less fluoride is ingested into the body. Foam types of fluoride agents are readily dispersed within the oral cavity and are easier to apply than other forms, even when patients have fixed orthodontic appliances present. Furthermore, *in vitro* research has shown no significant differences between APF gel and foam in the amount of uptake into enamel, and while foam could provide an effect similar to that of gel in terms of preventing dental caries [26, 27], there has not yet been any clinical research showing that the professional topical application of fluoride using 2% neutral NaF foam prevents dental caries. 

In this research, the number of subjects might not be enough large, it caused the large values of standard deviation in the results. Further research which included not only adults but also children as subjects is necessary to investigate the appropriate frequency of use and the effects in preventing dental caries.

## 5. Conclusions

The present results found the following:

1. When carrying out professional topical fluoride application using 2% neutral NaF foam with a dedicated tray, filling the tray with foam to a level approximately 2 mm below the tray edge was appropriate, and this amount made it possible to adequately cover all the dentition.
2. The appropriate amount of 2% neutral NaF foam used for permanent dentition was approximately 0.8 g on average, which was approximately one-fifth the amount of gel ordinarily used with the tray method.
3. The residual ratio of fluoride in the oral cavity following a 4-minute application of 2% neutral NaF foam was approximately 25% of the amount of foam used, and a strong correlation was observed between the amount of fluoride used per kilogram body weight and the amount of residual fluoride in the oral cavity per kilogram body weight.

## Figures and Tables

**Table 1:** The appropriate amounts of foam (AAF), tray-retained foam (TRF), overflowed foam (OF) and tooth-surface-retained (TSRF) on the deciduous dentition study model.

		AAF	TRF	OF	TSRF
Upper dentition	Mean	0.30	0.20	0.05	0.02
	SD	0.12	0.09	0.03	0.01
	Min.	0.12	0.07	0.01	0.00
	Max.	0.50	0.37	0.10	0.04

Lower dentition	Mean	0.27	0.12	0.13	0.02
	SD	0.12	0.05	0.08	0.01
	Min.	0.12	0.06	0.03	0.01
	Max.	0.44	0.21	0.29	0.03

Total	Mean	0.57	0.32	0.18	0.03
	SD	0.17	0.09	0.09	0.01
	Min.	0.36	0.20	0.09	0.01
	Max.	0.86	0.47	0.38	0.05
	C (%)	29.45	28.07	50.06	35.86

*n* = 10, unit: g, C: coefficient of variation.

**Table 2:** The appropriate amounts of foam (AAF), tray-retained foam (TRF), overflowed foam (OF) and tooth-surface-retained (TSRF) on the permanent dentition study model.

		AAF	TRF	OF	TSRF
Upper dentition	Mean	0.60	0.43	0.07	0.08
	SD	0.20	0.16	0.04	0.05
	Min.	0.30	0.15	0.02	0.02
	Max.	0.89	0.64	0.16	0.14

Lower dentition	Mean	0.35	0.18	0.11	0.04
	SD	0.16	0.06	0.10	0.02
	Min.	0.22	0.10	0.04	0.01
	Max.	0.75	0.32	0.38	0.06

Total	Mean	0.96	0.60	0.17	0.12
	SD	0.24	0.15	0.09	0.06
	Min.	0.68	0.34	0.10	0.03
	Max.	1.40	0.78	0.40	0.20
	C (%)	25.08	25.24	52.56	54.55

*n* = 10, unit: g, C: coefficient of variation.

**Table 3:** Coefficient of correlation between AAF and TRF, OF, and TSRF on deciduous and permanent dentition.

	AAF			
	Deciduous dentition		Permanent dentition	
	*r* value	*P* value	*r* value	*P* value
TRF	0.9282	.0001	0.8717	.0010
OF	0.9042	.0003	0.7914	.0064
TSRF	0.1680	.6426	0.4243	.2216

AAF: appropriate amount of foam, TRF: tray-retained foam, OF: overflowed foam, TSRF: tooth-surface-retained foam.

**Table 4:** The oral residual fluoride following the application of sodium fluoride foam.

subjects	Applied foam (g)	Applied fluoride (mg)	Total amounts of oral residual fluoride (mg)	Oral residual fluoride per kilogram body weight (*μ*g/kg)	Ratio of oral residual fluoride (%)
A	1.16	10.47	1.77	28.59	16.88
B	0.75	6.72	1.97	29.01	29.35
C	0.79	7.09	2.44	53.09	34.43
D	0.96	8.65	2.30	51.09	26.58
E	0.59	5.27	1.32	29.22	24.98
F	0.97	8.69	1.69	32.40	19.40
G	0.62	5.57	1.12	17.25	20.12
H	0.56	5.00	1.35	27.08	27.06

Mean	0.80	7.18	1.74	33.47	24.85
SD	0.22	1.94	0.47	12.32	5.80
C (%)	27.06	27.06	27.20	36.81	23.34

*n* = 8, C: coefficient of variation.
